# Refining developmental coordination disorder subtyping with multivariate statistical methods

**DOI:** 10.1186/1471-2288-12-107

**Published:** 2012-07-26

**Authors:** Christophe Lalanne, Bruno Falissard, Bernard Golse, Laurence Vaivre-Douret

**Affiliations:** 1 AP-HP, Department of Clinical Research, Saint-Louis Hospital, Paris, France; 2 Inserm Unit UMR-SO 669, University Paris Sud, Paris Descartes, Paris, France; 3 AP-HP, Paul Brousse Hospital, Public Health Department, Villejuif, France; 4 AP-HP, Necker-Enfants Malades Hospital, Paris, France; 5 University Paris Descartes, Sorbonne Paris Cité, France; 6 AP-HP, Port Royal-Cochin Hospital, Dept. Obstetrics & Gynecology, Paris, France

## Abstract

**Background:**

With a large number of potentially relevant clinical indicators penalization and ensemble learning methods are thought to provide better predictive performance than usual linear predictors. However, little is known about how they perform in clinical studies where few cases are available. We used Random Forests and Partial Least Squares Discriminant Analysis to select the most salient impairments in Developmental Coordination Disorder (DCD) and assess patients similarity.

**Methods:**

We considered a wide-range testing battery for various neuropsychological and visuo-motor impairments which aimed at characterizing subtypes of DCD in a sample of 63 children. Classifiers were optimized on a training sample, and they were used subsequently to rank the 49 items according to a permuted measure of variable importance. In addition, subtyping consistency was assessed with cluster analysis on the training sample. Clustering fitness and predictive accuracy were evaluated on the validation sample.

**Results:**

Both classifiers yielded a relevant subset of items impairments that altogether accounted for a sharp discrimination between three DCD subtypes: ideomotor, visual-spatial and constructional, and mixt dyspraxia. The main impairments that were found to characterize the three subtypes were: digital perception, imitations of gestures, digital praxia, lego blocks, visual spatial structuration, visual motor integration, coordination between upper and lower limbs. Classification accuracy was above 90% for all classifiers, and clustering fitness was found to be satisfactory.

**Conclusions:**

Random Forests and Partial Least Squares Discriminant Analysis are useful tools to extract salient features from a large pool of correlated binary predictors, but also provide a way to assess individuals proximities in a reduced factor space. Less than 15 neuro-visual, neuro-psychomotor and neuro-psychological tests might be required to provide a sensitive and specific diagnostic of DCD on this particular sample, and isolated markers might be used to refine our understanding of DCD in future studies.

## Background

Neuropsychological and psychiatric studies often involve a large collection of testing instruments, each aiming to assess more or less specific facets of one’s behavorial and psychological profile. The number of available cases appears rather small (*n*<60) in some cases, due to the low prevalence of the outcome of interest and/or costs associated to data collection. In such a situation, it becomes critical to select the most relevant items to the study at hand which amounts to find a good compromise between screening efficacy or diagnostic accuracy, consulting time, and availability of dedicated testing batteries. Another concern is that researchers typically want to assess what best characterize clinical subgroups and how homogeneous they are. The present study aims at performing *feature extraction*, that is selecting the most informative items, when diagnosing dyspraxia in children during planned clinical examination. A second objective is to show that there exist specific impairments that are relevant and consistent within clinical subgroups; in other words, we seek to build a *typology of the patients*.

### Clinical subtyping of developemental coordination disorder

With a prevalence up to 10% worldwide (higher in boys), developmental coordination disorder (DCD) constitutes a major challenge from a public health perspective as it may lead to learning difficulties, behavioral disorder, or social and emotional maladaptation. Dyspraxic patients are usually screened based on their impairments in motor coordination or usual visuo-motor and neuropsychological tests batteries [1,2], and are often categorized as patients suffering from DCD [3]. However, the DSM-IV-R criteria remain vague with regard to the exact nature of those impairments and the relevance and consistency of dyspraxia subtyping within DCD category. Previous approaches mainly relied on cluster analysis to refine the distinction, although the number of reported subtypes generally varied between three and six [2,4-8]. This heterogeneous clustering is attributable in part to the difference in the testing material (e.g., BruininksOseretsky Test of Motor Proficiency, BOTMP, or Movement Assessment Battery for Children, M-ABC) used in these studies, but more importantly to the fact that they largely focused on coordination and motor performance in relation to learning. As pointed out by Wilson [9], a normative functional skill approach suffers from the selection of tasks that are not necessarily representative of the various facets of motor control and movement skills, such that “a multi-level approach to assessment and treatment is recommended for children with DCD. The use of multiple and converging measures will circumvent existing issues with diagnosis and promote a fuller appreciation of motor development at different levels of function–behavioural, neurocognitive, and emotional” (p. 819).

In a recent study, Vaivre-Douret and coll. [10] provided a more detailed account of children exhibiting different types of sensory-motor deficit by using a broader testing battery. These authors systematically assessed academic, language, cognitive, visual-spatial, and visual-motor perception skills, while using additional standardized neuro-developmental psychomotor tests, including motor coordination, neuro-visual, and neuromuscular tone examination. It was concluded that ‘pure’ forms of developmental dyspraxia—ideomotor and visual-spatial/visual-constructional—may be distinct from specific motor coordination disorder, and more frequently associated to various neuropsychological disorders and soft neurological signs. A ‘mix’ group exhibiting specific motor coordination disorders with a large number of learning disorders was isolated from these two ‘pure’ forms. Moreover, it was suggested that motor planning and programming appear to be the core problem underlying children difficulties, and not performance *per se*. The implications of these findings from an etiological standpoint goes beyond the scope of the present article, and the interested reader is referred to the above article for a more in-depth discussion. The above results will be used to refine neuro-visual, neuro-psychomotor and neuro-psychological markers that are characteristic of this three-subtype classification.

### Statistical approaches for feature extraction

A crucial aspect of explanatory statistical inference in this context is that we need methods that allow to deal with categorical outcomes and to weigh a large number of potentially correlated predictors while preventing from overfitting. We will here focus on two multivariate statistical techniques that seem to meet these two criteria.

As an extension to classification and regression trees (CART), Leo Breiman proposed the *Random Forests* (RF) algorithm which retains many benefits of decision trees while achieving better results and competing with penalized SVM, Neural Networks or Gradient Boosting Machines [11,12]. The RF algorithm is built upon the general framework of Bagging [13]: It relies on resampling via the boostrap procedure but add an extra randomization step at the level of the variables. As such it overcomes the limitations of linear classifiers and yield an ensemble of unpruned trees that achieve a good balance between bias and variance.

Another method which might also be applied with a low ratio of samples (*n*) to potentially correlated variables (*p*) is Partial Least Square Discriminant Analysis (PLS-DA). This is a regression method that seeks to sharpen the separation between groups of observations while constructing maximally covarying linear combinations of the original predictors. It has been successfully used in proteomic studies [14] or microarray expression data [15]. Although PLS regression might be directly applied when the number of variables *p* is greater than the number of observations *n*, several methods for variable ranking [16,17] and selection [18,19] have been proposed (for a review, [20]), it is also possible to consider a more parcimonious model by adding constraints during parameter estimation. Regularization or so-called “shrinkage” methods consider a weighted variance-covariance matrix, as in ridge regression [21]. While reducing their variance, it also increases the bias of the parameter estimates. An alternative penalization scheme is the *elastic net* criterion proposed by Zou and Hastie [22]. Following their notations, it is defined as the argument that minimizes, over the vector of parameters *β*, the following loss function:

(1) $$L(\lambda_{1},\lambda_{2},\beta )=∥Y-\mathrm{X\beta}{∥}^{2}+\lambda_{2}∥\beta {∥}^{2}+\lambda_{1}∥\beta {∥}_{1},$$

where $∥\beta {∥}^{2}=\overset{p}{\underset{j=1}{\sum }}\beta_{j}^{2}$ and $∥\beta {∥}_{1}=\overset{p}{\underset{j=1}{\sum }}|\beta_{j}|$. The *λ*’s are the penalty parameters. This combination of *L*_1_ and *L*_2_-norm penalties achieves both shrinkage and automatic variable selection, while allowing to keep *m*>*n* variables in the case where *n*≪*p*. Chun and Keleş [23] considered this kind of penalization for sparse PLS regression, based on the SIMPLS algorithm [24], although by setting $\lambda_{2}=\infty$ there remains only two tuning parameters, the number of hidden components *K* and the thresholding parameter *λ*_1_. An alternative formulation of Lasso (*L*_1_) penalization was proposed by Lê Cao and coll. in related work [25]; specifically, the penalization now takes the form of a soft-thresholding rule applied on variable loadings during the iterative steps of the NIPALS algorithm [26].

In addition to protect against increased false positive rate arising from multiple comparisons in univariate screening of interesting predictors, such embedded methods have been proved to compete with wrapper methods [27,28], and a recent study showed that sparse PLS and RF provide sensible and interpretable results with gene expression data [29].

The rest of this article is organised as follows: participants and clinical assessment are described first, together with the estimation of model parameters and measures of variable importance; then we present the results obtained with RF and unpenalized or penalized PLS-DA; finally, these results are discussed in the context of DCD subtypes identified in [10].

## Methods

### Participants and testing material

The data are comprised of a set of *N* = 63 children (5 to 15 years old with a median age of 8.1 yrs., 83% of males). Patients were enrolled based on DSM-IV-R criteria: mild to moderate motor-coordination difficulties interfering with the performance of daily activities (criterion A), and with academic achievement (criterion B). They were free of previous assessment, and no comorbidities (e.g., ADHD, neurological disorder, visual or auditory deficit) were detected during first examination.

Following clinical examination detailed in [10], all patients were classified as suffering from either ideomotor (IM), visual-constructional and spatial (VSC), or mixt (MX) dyspraxia. For each subject, binary-scored responses (0=success, 1=failure) based on percentile or SD thresholds were available for a set of 49 items covering visual, motor, perceptuo-motor, and general performance.

Neuro-psychological assessment consisted in administering subtests of a standard Wechsler measure of intelligence, and standardized tests of visual constructional skills (block design), visual-spatial structuring (Rey’s geometric figures and Beery’s Visual-Motor Integration test), visual-spatial attention (bell-crossing test), mental executive functions (Porteus Labyrinth and Tower of London test). A handwriting scale was also used to detect dysgraphy, visual perception was assessed with form recognition tasks, and kinaesthetic perception (memory) was assessed by positioning child’s arm and finger and asking him with eyes closed to remember and repeat. A language screening battery included tasks of reading, repetition of words and logatoms, picture-naming speed, meta-phonological tests, auditory memory and working memory tasks (digit span). Neuro-psychomotor assessment was based on the “neuro-psychomotor functions in children” battery (NP-MOT), which allows to measure developmental maturation of the following functions: neuromuscular examination, gross motor-control tasks, laterality, praxis, digital gnosis, manual dexterity, body spatial integration, rhythmic tasks, auditory-attentional task [30]. Finally, neurovisual examination included electro-retinogram, visually evoked potentials and motor electro-oculogram.

For clarity purpose, the full set of items has been abbreviated using four-letter acronyms (see List of abbreviations used).

This study was conducted by Inserm Unit 669 in the out-patient consultation of the Child Psychiatry Department, Necker Hospital, Paris. Institutional review board approval was obtained for the clinical investigations, and this study is in compliance with the ethical principles for medical research as presented in the Helsinki Declaration. Written informed consent was obtained from the participant (parents and children) for publication of this report and any accompanying images.

### Statistical models

The RF algorithm can be summarized as follows. Given ntree number of trees to grow and mtry variables used to split each node: 

1. Construct a bootstrap sample of size *n*<*N*, with replacement, and start growing a tree for this sample.

2. When growing the tree, use mtry variables selected at random to find the best split.

3. Repeat the preceding step until the tree reaches its maximal extent (no pruning).

Each observation is classified using the principle of majority voting after having collected votes from every trees in the forest. A realistic measure of predictive accuracy can be obtained by using so-called out-of-bag (OOB) samples, which amounts to about one third of the individuals not considered when growing each tree since bootstrap with replacement is used. In addition, a built-in permutation-based measure of variable contribution to prediction accuracy allows to rank variables by their importance. The number of times individuals from the training and OOB samples are found to belong to the same terminal node can be used as a measure of their ‘likeness’, hence the measure of pairwise proximity, appropriately normalized by the number of trees, that can be used to cluster individuals using traditional metric dimensional scaling (MDS). It is worth noting that irrelevant descriptors will have little influence on this proximity measure.

The PLS-DA classifier consists in a classical PLS regression [26,31] where we seek to construct from the explanatory block *X* (of dimensions *n*×*p*) a set of *K* orthogonal orthogonal factors scores or latent variables, *ξ*_1_,…,*ξ*_*K*_, with associated loadings, *u*_1_,…,*u*_*K*_, that maximize the covariance between *X* and an univariate or multivariate response block *Y *. Let *Y * be a single vector of outcomes, this yields the following optimization problem:

(2) $$\underset{||u_{k}||=1}{\max}\text{cov}(X_{k-1}u_{k},Y),$$

where *X*_*k*−1_ is the residual matrix in the regression of *Y * on *ξ*_*k*_=*X*_*k*_*u*_*k*_, for each component *k*=1,…,*K*. The sign and magnitude of the *u*_*k*_’s give an indication about the contribution of each variable in the construction of the components scores, *ξ*_*k*_. In PLS-DA, the categorical outcome of interest *Y * is recoded in a set of dummy variables expressing individual class membership. Considering *C* classes, we define an indicator matrix *Z* based on *Y *

(3) $$Z_{c}=\left\{\begin{array}{ll} 1 & \text{if}\;Y=y_{c},\\ 0 & \text{otherwise}, \end{array}\right)$$

and construct *C* classification functions of the form

(4) $${\hat{Z}}_{c}=b_{0,c}+b_{1,c}X_{1}+\cdots +b_{p,c}X_{p},\;c=1,\ldots ,C,$$

where the *b*_*i,c*_’s are the regression coefficients asssociated to the *c*th class.

### Model calibration

The sample was divided into a training sample and a validation sample, using a split ratio of 0.7/0.3. Model building and feature extraction were performed on the training sample only. The validation set was used to assess the predictive power of the models and clustering fitness.

Tuning of hyperparameters for RF (number of variables used to build a single tree, mtry) and PLS-DA (number of dimensions, *K*, and/or sparsness parameter, *η*) was done using a nested cross-validation scheme, comprised of stratified and repeated 10×5-fold resampling (inner loop) combined to a search grid of length 10 for the hyperparameters (outer loop). The number of trees considered in RF was kept constant (ntree=500). For sparse PLS-DA, we used a custom grid of tuning parameters with 10 uniformly spaced 0.3<*η*<0.9, for *K* ranging from 1 to 10. The criterion to select model parameter(s) was the average classification accuracy computed on hold-out samples across resampling results. Accuracies were compared between models using the method proposed in [32].

### Variables scoring

For RF, we considered the mean decrease in accuracy to assess variable importance. For PLS-DA, items loadings were used as overall (i.e., not class-specific) measures of variable importance for each of the extracted component. In both cases, the significance of all measures of variable importance was tested using a permutation strategy, whereby class labels were randomly exchanged and variable importance was recomputed on a total of 999 samples. For sparse PLS-DA, only 95% bootstrap confidence intervals associated to regression coefficients were computed.

**Table 1:** Descriptive statistics for the training and validation samples


		**Training**	**Validation**	**Combined**
	**N**	***N*** **=46**	***N*** **=17**	***N*** **=63**
Diagnosis: IM	63	9% ( 4)	6% ( 1)	8% ( 5)
VSC		52% (24)	53% ( 9)	52% (33)
MX		39% (18)	41% ( 7)	40% (25)
Gender: Male	63	78% (36)	94% (16)	83% (52)
Age (years)	63	6.8 8.0 9.7	6.6 8.7 12.3	6.8 8.1 10.4
Term: Yes	63	96% (44)	88% (15)	94% (59)
FIQ	62	85 98 114	92 108 121	86 100 115
PIQ	62	73 87 102	75 93 107	74 90 105
VIQ	62	92 107 122	100 119 130	92 110 124

Three-number summaries are lower quartile, median, and upper quartile.

*N* is the number of non–missing values.

### Predictive accuracy

For the *training sample*, prediction of class membership was based on the internal voting scheme for RF, whereas for PLS-DA a softmax method was used, whereby the predicted class, *c*^∗^, is the largest class probability after model predictions have been transformed on a [0,1] interval (with unit sum), that is

(5) $$Y=y_{c}\;\text{where}\;c^{*}={arg max}_{0\leq Z_{c}\leq 1,\sum Z_{c}=1}({\hat{Z}}_{c}).$$

For the *validation sample*, we computed classification accuracy based on the optimized model parameters.

### Clustering fitness

The PAM algorithm[33] was used to identify one representative sample (“medoid”) for each cluster, based on the PLS components scores in the training sample. The number of clusters was determined by maximizing the overall average silhouette width (ASW). The stability of the resulting partition was assessed using the bootstrap procedure described in [34]: For each bootstrap sample, Jaccard similarities between the original three-cluster solution and the one found on resampled data were averaged clusterwise. In addition, we verified whether cluster might be considered as isolated clusters (L- or L^∗^-cluster) or not. According to [33], a cluster is an L^∗^-cluster if and only if its diameter is smaller than its separation. A cluster is an L-cluster if and only if for each observation *i* the maximal dissimilarity between *i* and any other observation of the cluster is smaller than the minimal dissimilarity between *i* and any observation of another cluster.

Cluster affinity was defined as the euclidean distance between each observation in the validation sample and its expected cluster medoid. This mimic the isolation measure described above, though it is based on a distance and not a similarity measure.

### Statistical software

All analyses were performed with the open-source software, version 2.12 [35], the randomForest, pls and spls packages, and the caret interface for machine learning [36]. Group comparisons were performed at a fixed 5% Type I risk level, with correction for multiple comparisons (Bonferroni method) when justified.

## Results

### Patients characteristics

The main patients’ characteristics, including clinical diagnosis, for the training (*n* = 46) and test (*n* = 17) samples are shown in Table 1. As described in the original article [10], patients were mostly 8 years old males, with full IQ in the expected range. Nine cases out of ten were diagnosed as suffering from either VSC or MX dyspraxia, whereas only five subjects were classified as IM-dyspraxic.

Interitem Pearson correlations were in the range [-0.411;0.831] (median, 0.087). The marginal proportions of item failure were between 7.9% (Sitting alone) and 92.1% (Visual motor integration).

**Figure 1:**
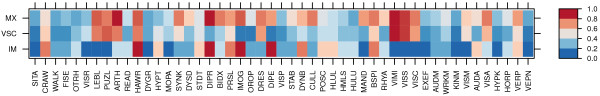
Conditional frequencies of impairment on all items.

Item failures for the whole cohort are summarized in Figure 1 as a heatmap where higher relative frequencies of failure are indicated in red. As can be seen, there are systematic patterns of failure that are clearly visible for some groups, for example digital praxia (DIPR) in MX and IM patients, arithmetic (ARTH) in MX patients only, visual-motor integration (VIMI) in MX and VSC patients, or digital perception (DIPE) in IM patients only. Also, there are some evidence for covarying items scores: IM patients were not impaired on lego (LEBL) and puzzles (PUZL) tasks, nor any visuo-motor tasks (VIMI, VISS, VISC), whereas VSC patients show systematic failures on the latter.

The average level of success did not differ between the training and test samples on any of the studied variables (all *p*>0.05, with *p*-values computed from Monte Carlo *χ*^2^significance tests).

### Model calibration

#### Random forest

The number of variables retained for growing trees was estimated at mtry=12, yielding an optimal classification accuracy of 0.924 (SD 0.055). Of note, this value is near the recommended default value for this parameter ($\sqrt{49}=7$).

For the final model, the OOB estimate highlighted an error rate of 8.7%, with 2 VSC (8.3%) and 2 MX (11.1%) missclassified patients on the training sample. An informal look at the evolution of error rates as a function of the number of trees indicated that the OOB error was stabilized after 225 trees were grown.

#### PLS-DA

For standard PLS-DA, six components were selected for an average classification accuracy of 0.917 (SD 0.088). For penalized PLS-DA, the optimal parameters were found to be *K* = 2 components and *η*=0.7 for sparseness. This resulted in a classification accuracy of 0.942 (SD 0.076), with only one missclassified VSC patient (4.2%). It should be noted that these two classification accuracies do not differ one from the other (*p* = 0.346, with Bonferroni correction), nor with classification accuracy estimated for RF (*p* = 0.491, for sPLS-DA).

### Variable importance

#### Random forest

**Figure 2:**
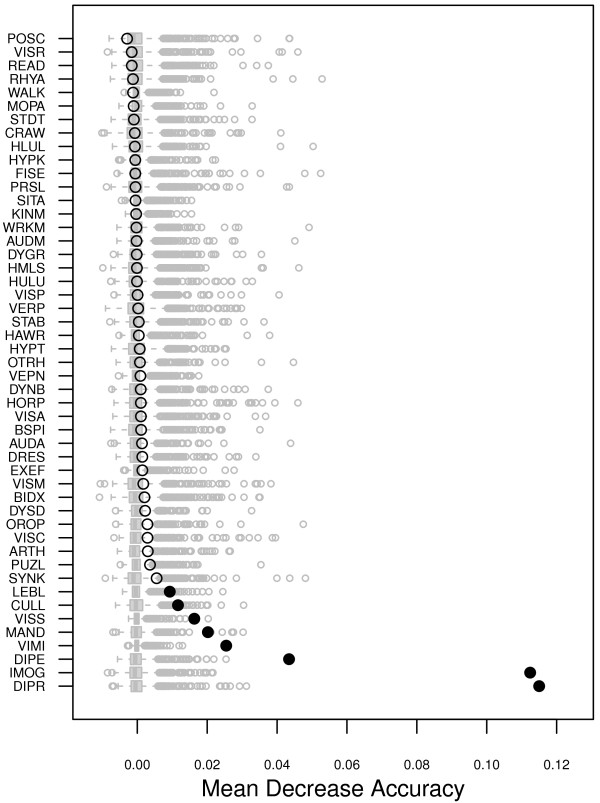
Scree plot of the measures of variable importance in RF.

The importance of variables in RF, as measured by the mean decrease accuracy, are shown in Figure 2. The original estimates from the retained model during parameters tuning are shown as black circles, and the importance computed through re-randomization are shown as Tukey’s boxplots in grey color. Filled symbols indicate a significant permutation test at the 5% level. In this case, eight variables showed a consistent and significant contribution to overall accuracy on the training sample. These are, in decreasing order of magnitude: digital praxia (DIPR), imitation of gestures (IMOG), digital perception (DIPE), visual motor integration (VIMI), manual dexterity (MAND), visual spatial structuration (VISS), coordination between upper and lower limbs (CULL), and lego blocks (LEBL). Class-specific measures of variable importance are also provided in Table 2.

**Table 2:** Class-specific measures of variable importance for RF, PLS-DA and sPLS-DA


	**RF**			**PLS**			**sPLS**		
	**IM**	**VSC**	**MX**	**IM**	**VSC**	**MX**	**IM**	**VSC**	**MX**
SITA	0.17	0.14	0.15	0.01	0.10	0.16	—	—	—
CRAW	0.17	0.12	0.18	0.04	0.06	0.19	—	—	—
WALK	0.11	0.18	0.10	0.03	0.06	0.18	—	—	—
FISE	0.12	0.20	0.12	0.04	0.18	0.17^4,6^	—	—	—
OTRH	0.15	0.13	0.27	0.07	0.06	0.17^3,6^	—	—	—
VISR	0.31	0.13	0.09	0.20	0.24	0.19	—	—	—
LEBL	0.72	0.23	0.26^⋆^	0.35	0.16	0.20	0.96	0.03	0.03 —
PUZL	0.64	0.16	0.10	0.36	0.21	0.18^2^	0.96	0.09	0.09 —
ARTH	0.18	0.14	0.34	0.11	0.20	0.24	—	—	—
READ	0.16	0.16	0.09	0.13	0.22	0.21^6^	—	—	—
HAWR	0.31	0.20	0.16	0.02	0.04	0.12^5^	—	—	—
DYGR	0.17	0.20	0.12	0.02	0.02	0.04^4^	—	—	—
HYPT	0.08	0.24	0.14	0.01	0.31	0.32	—	—	—
MOPA	0.17	0.12	0.14	0.32	0.32	0.40	—	—	—
SYNK	0.54	0.13	0.28	0.25	0.18	0.23	0.81	0.16	0.16 —
DYSD	0.30	0.22	0.22	0.30	0.37	0.39	—	—	—
STDT	0.06	0.15	0.15	0.16	0.04	0.15^3^	—	—	—
DIPR	0.75	0.58	0.67^⋆^	0.45	0.98	0.96	0.91	0.00	0.91 —
BIDX	0.09	0.16	0.25	0.07	0.42	0.39	—	—	—
PRSL	0.11	0.18	0.16	0.18	0.18	0.28	—	—	—
IMOG	0.79	0.60	0.65^⋆^	0.55	0.99	1.00	1.00	0.88	0.88 —
OROP	0.36	0.22	0.28	0.03	0.27	0.33	—	—	—
DRES	0.28	0.23	0.19	0.21	0.09	0.06	—	—	—
DIPE	0.69	0.48	0.39	0.35	0.72	0.59	0.96	0.67	0.67 —
VISP	0.14	0.20	0.16	0.00	0.13	0.16	—	—	—
STAB	0.30	0.17	0.16	0.14	0.25	0.20	—	—	—
DYNB	0.18	0.20	0.20	0.11	0.25	0.47^3^	—	—	—
CULL	0.50	0.29	0.27^⋆^	0.11	0.53	0.61	0.81	0.59	0.59 —
POSC	0.08	0.12	0.07	0.11	0.04	0.21^3,5^	—	—	—
HLUL	0.00	0.17	0.16	0.14	0.06	0.09	—	—	—
HMLS	0.09	0.15	0.19	0.08	0.10	0.06	—	—	—
HULU	0.11	0.19	0.17	0.09	0.04	0.04	—	—	—
MAND	0.56	0.31	0.41^⋆^	0.10	0.57	0.66	0.81	0.64	0.64 —
BSPI	0.17	0.22	0.22	0.10	0.08	0.11	—	—	—
RHYA	0.13	0.10	0.17	0.13	0.26	0.30	—	—	—
VIMI	1.00	0.30	0.42^⋆^	0.39	0.15	0.27^2^	0.00	0.00	0.00 —
VISS	0.94	0.34	0.17^⋆^	0.39	0.20	0.27^2^	0.99	0.06	0.00 —
VISC	0.52	0.22	0.17^⋆^	0.31	0.14	0.14^2^	0.87	0.09	0.09 —
EXEF	0.24	0.25	0.16	0.07	0.27	0.26	—	—	—
AUDM	0.10	0.15	0.18	0.19	0.05	0.18^4^	—	—	—
WRKM	0.24	0.14	0.17	0.23	0.10	0.12^4^	—	—	—
KINM	0.17	0.12	0.15	0.10	0.03	0.08^3,6^	—	—	—
VISM	0.38	0.24	0.18	0.34	0.14	0.08^2^	—	—	—
AUDA	0.17	0.24	0.15	0.17	0.27	0.34	—	—	—
VISA	0.24	0.16	0.19	0.16	0.21	0.23	—	—	—
HYPK	0.17	0.14	0.10	0.13	0.10	0.14^5^	—	—	—
HORP	0.08	0.26	0.14	0.09	0.23	0.29	—	—	—
VERP	0.11	0.20	0.19	0.09	0.16	0.21	—	—	—
VEPN	0.17	0.23	0.17	0.00	0.14	0.21	—	—	—

^⋆^denote significant measure of variable importance in the PLS case, and upper script numbers indicate on which PLS component a variable was found significant at the 5% level.

#### PLS-DA

For PLS-DA, the following important variables were found, in decreasing order of magnitude (items found on more than one component are emphasized in italic letters): **(Component 2)** visual spatial memory (VISM), puzzles (PUZL), visual spatial constructional (VISC), visual spatial structuration (VISS), lego blocks (LEBL), visual motor integration (VIMI); **(Component 3)***postural control* (POSC), dynamic balance (DYNB), standing tone (STDT), *kinaesthetic memory* (KINM); **(Component 4)** work memory (WRKM), auditivo memory (AUDM), *first sentences* (FISE), dysgraphia (DYGR); **(Component 5)***postural control* (POSC), hand writing (HAWR), hyperkinesia (HYPK) **(Component 6)** reading/spelling (READ), *kinaesthetic memory* (KINM), *first sentences* (FISE), otorhinolaryngologia (OTRH). It should be noted that none of the variables reach the 5% significance level on the first component. Class-specific loadings are summarized in Table 2.

**Figure 3:**
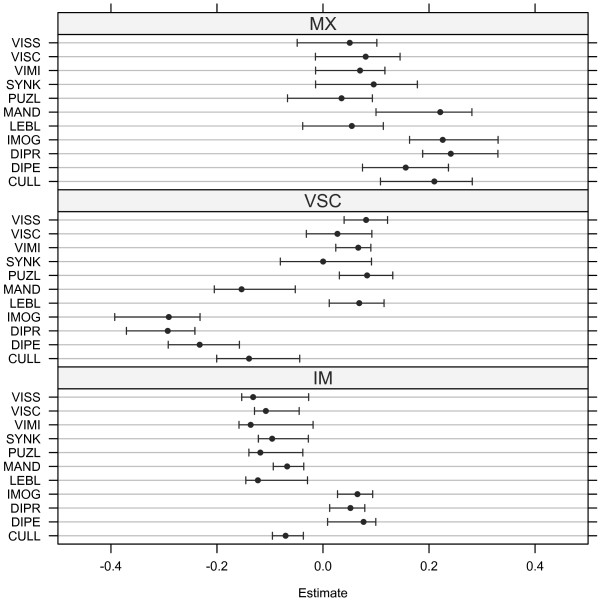
**Sparse PLS-DA regression coefficients with associated 95% confidence intervals computed using***B***=1,000 bootstrap samples.**

On the contrary, eleven variables were selected by sPLS-DA: lego blocks (LEBL), puzzles (PUZL), synkinesia (SYNK), digital praxia (DIPR), imitation of gestures (IMOG), digital perception (DIPE), coordination between upper and lower limbs (CULL), manual dexterity (MAND), visual motor integration (VIMI), visual spatial structuration (VISS), and visual spatial constructional (VISC). This set of variables closely matched the one outlined with RF method, and is a subset of the variables with highest loadings for the unpenalized PLS-DA approach. Variables loadings are given in Table 2 and regression coefficients with their associated 95% confidence intervals are displayed in Figure 3.

### Predictive classification accuracy

Classification accuracy on the validation sample was perfect in the case of RF, and identical for PLS and sPLS (0.941, 95% CI [0.713;0.999]), with only one VSC patient missclassified.

### Projection of individuals in the feature space

**Figure 4:**
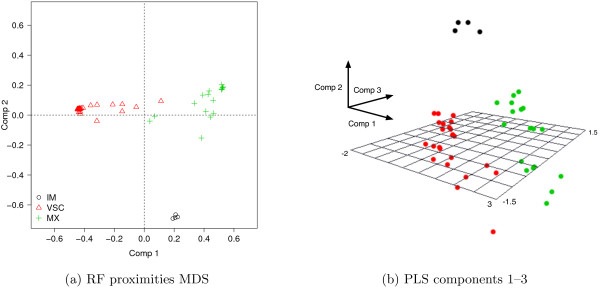
Plots of individual factor scores computed from (a) the RF proximities matrix and (b) PLS loadings.

Figure 4 shows individual locations in a reduced factorial space defined by multidimensional scaling applied to individual proximities computed from RF (Figure 4a), and projection of factor scores in the first three dimensions of PLS-DA (Figure 4b).

In the case of PLS-DA, the first component is determined by an opposition between hypotonia (high negative loading) and manual tasks (imitation of gestures, digital praxia, digital perception, manual dexterity). The second axis is mainly driven by the same manual tasks, except manual dexterity, vs. visuo-spatial tasks (puzzles, visual spatial structuration, visual spatial memory). On the third axis, the same visuo-spatial and manual tasks have high negative loadings while dynamic balance, motor pathways and auditivo memory have high positive values.

### Patients typology

With component scores computed from the PLS-DA model, the optimal number of clusters was estimated at three, with an average silhouette width of 0.348. Although this is indicative of a weak clustering structure, the cross-classification of cluster and diagnosis classes was satisfactory: Two VSC patients were considered as belonging to the cluster composed of MX patients only (*n* = 18). When using bootstrap (500 samples), the clusterwise Jaccard similarity values were all above 0.5, except for the smaller cluster (Table 3).

**Table 3:** Measures of predictive accuracy and clustering fitness


**Classifier**	**Class**	**Sensitivity**	**Specificity**	**ASW**	**Isolation**	**Jaccard**	**C1**	**C2**	**C3**
PLS-DA	IM	1.00	1.00	0.625	L^*^	0.462	**1.587**	2.784	1.206
	VSC	0.89	1.00	0.369	No	0.665	3.106	**0.838**	2.315
	MX	1.00	0.90	0.270	No	0.605	2.811	2.416	**0.587**
sPLS-DA	IM	1.00	1.00	1.000	L^*^	0.792	**0.129**	0.437	0.386
	VSC	0.89	1.00	0.712	No	0.928	0.468	**0.103**	0.282
	MX	1.00	0.90	0.479	No	0.854	0.487	0.330	**0.073**

L or L^*^ denotes isolated cluster (See text for details).

For the penalized PLS-DA model, three clusters were identified by optimizing the average sihouette width (0.625). The clusterwise Jaccard bootstrap measures were all in the acceptable range (≥0.8), and 4 VSC patients were found in the cluster composed of MX patients.

Except for the minority cluster (IM), the representative individuals were different in the two PLS models.

### Clustering fitness

**Figure 5:**
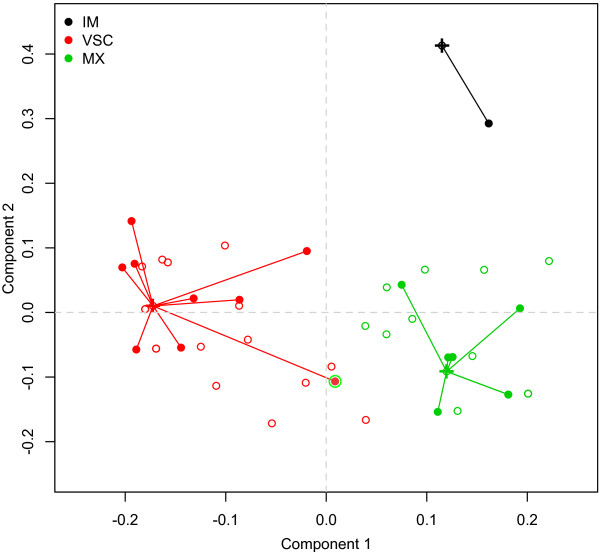
**Individual coordinates from sPLS-DA for the training (open circles) and validation (filled circles) sample.** The medoids for each cluster are shown using a cross.

As can be seen in Table 3, the average euclidean distance of patients from the validation sample to their expected medoids (C1 to C3) was always less than the average distance to other medoids, except for IM patients with PLS-DA. When considering sPLS-DA, the MX group appears to exhibit more compactness since it has the lowest average distance measure. This is further illustrated in Figure 5 which shows patients’ location in the factor space defined by the two components of the sPLS-DA model. The misclassified individual has been highlighted using a double circle.

### Pattern of association between selected variables and clinical diagnosis

**Figure 6:**
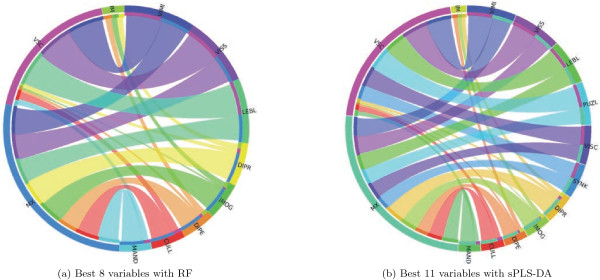
Association between clinical group and variables ranked or selected as most important in RF and sPLS-DA.

The conditional and marginal distributions of item failure by clinical subgroup is summarized using a circular tabular display (Circos, http://mkweb.bcgsc.ca/tableviewer) in Figure 6, considering variables selected by RF and sPLS-DA on the training sample. The size of each ribbon reflects the strength of the association (i.e., cell counts in the corresponding 7 or 11×3 table), while the outer segments indicate marginal frequencies. Such a picture offers an intuitive visualization of the following three main characteristics of task failure due to specific dyspraxia: (a) IM patients are equally impaired on digital perception (DIPE), imitations of gestures (IMOG), and digital praxia (DIPR), (b) some items are commonly found in both VSC and MX patients, that is lego blocks (LEBL), visual spatial structuration (VSS), and visual motor integration (VIMI), whereas (c) some items remain mostly specific of MX, and to a lesser extent VSC dyspraxia, namely digital praxia, imitation of gestures, and more importantly coordination between upper and lower limbs (CULL) and digital perception (DIPE).

Of the 11 items isolated with sPLS-DA, IMOG and DIPE were found significantly associated with clinical diagnosis in the validation sample at a 5% Bonferroni-corrected level (0.05/11=0.0045). The *p*-values for digital praxia and synkinesis were below 10%.

## Discussion

The primary aims of this article were to determine the most relevant items for distinguinshing between three DCD subtypes, and to quantify the homogeneity of patients within each subtype. Two multivariate methods, RF and PLS-DA, were shown to be useful to select the most informative items from a large set of testing instruments with high sensitivity and specificity, while allowing to characterize a set of 63 patients from a multivariate perspective. Imposing sparsity when building PLS components led to more direct and interpretable results.

### Interest of multivariate classification

Feature selection based on RF has been proposed in the past, including the use of permutation techniques. For example, Diaz-Uriarte and Alvarez de Andrés [37] proposed a backward elimination algorithm to select relevant subset of genes based on variable importance. Using this method, as implemented in the varSelFR R package, with a slight different configuration for RF (500 trees, but with the mtry parameter set at its default value of $\sqrt{p\;}$), five variables were selected: digital perception (DIPE), digital praxia (DIPR), imitation of gestures (IMOG), manual dexterity (MAND), and visual motor integration (VIMI). The .632+ Bootstrap estimate of prediction error was found to be 0.0713 (using 500 replicates), which is in close agreement with the prediction error observed on our training sample. It should be noted, however, that permuting clinical labels allows to verify the existence of a class structure in the dataset, not whether the classifier truly exploits items dependency [38].

Contrary to Robert-Granié et al.’s study [29], our results didn’t show a clear improvement of sparse PLS over unpenalized PLS when predicting diagnostic classes, although they both yielded a consensual subset of important variables. This might be explained by the high signal-to-noise ratio for some of the neuro-psychological tests used in this study.

Another point that deserves some discussion concerns the choice of the metric used to quantify variable importance in PLS-DA. In this study, variable loadings were used as they reflect the “weight” of the variables when building component scores that maximize the discrimination among classes. Other measures of variable importance have been proposed, for example Variable Importance in Projection (VIP), but see [20] for a review. We found, however, that using VIP-based measures of variable importance yielded results in close agreement with the one reported in this study.

Also, random forests is a nonparametric approach that supports multivariate and nonlinear associations whereas PLS regression models linear dependencies only, which may yield different variable importance measures especially in the case where highly nonlinear associations between predictors of interest are present. A combination of the two approaches, where either RFs [39] or PLS [40] is used to perform dimensionality reduction, has been successfully applied in some domains. Menze et al. [41] demonstrated that on spectral data univariate feature screening will perform poorer than multivariate Gini importance computed from RFs which in turn is able to highlight higher-order interaction effects. However, PLS-DA was found to perform better overall for classification, as compared to RFs. The superiority of PLS-based classifiers was also confirmed in presence of additive global noise on synthetic datasets, but its performance decreased when irrelevant features were added. Nevertheless, recursive feature elimination based on Gini importance can be used to remove features with non-discriminatory variance before applying a PLS-DA classifier. This suggests that depending on the structure of the data under consideration, a combined approach where RFs are used to perform dimensionality reduction and some form of regularization on input data before they enter a linear classifier or projection to latent structures might a be viable alternative. Other interesting approaches have been proposed as well, for example Logic Regression [42] which also relies on the idea of bagging boolean trees to identify significant interactions among a set of descriptive binary variables, see also [43,44].

Finally, RF and PLS-DA provide efficient ways for visualizing how patients and variables cluster together when considering all variables at the same time (unlike univariate screening approaches), which has already been discussed by [45]. They both appear to nicely complement each other. Looking at patients’ locations in the PLS factorial space leads to a more direct interpretation of the relationships between subjects and variables, or between variables themselves, since the latent dimensions extracted from PLS-DA are just linear combinations of the original variables. On the other hand, screening variables of interest through RF is relatively straightforward, whereas relying on PLS-DA often means “reading” beyond the first dimension. For example, RF considered digital praxia and imitation of gesture as the two most important variables, whereas they were found on separate dimensions when using PLS-DA.

### Clinical implications

The present findings are consistent with the previous observation that difficulties in planning and programming movement, rather than executive disorders, might partly be responsible for the observed typology in this sample of 63 children.

Indeed, our results confirmed the importance of some aspects of visual processing of spatial information and motor control in developmental coordination disorder and their subtle association in delineating DCD subtypes, as discussed in [10]. Digital praxia and imitation of gestures help distinguishing between visuo-constructional and spatial dyspraxia (no impairment) and ideomotor or mixt dypraxia, whereas visual motor integration and visual spatial structuration are more characteristic of the opposition between ideomotor dyspraxia (no impairment) and the two other subtypes. Hence, mixt dyspraxia is characterized by the presence of disorders specific of VSC or IM dyspraxia, but further includes unique comorbidities such as problem in coordinating upper and lower limbs, poorer manual dexterity or synkinesia which could be specific markers of developmental coordination disorder.

When assessing only performance on motor coordination in relation to learning development, it is likely that we would fail to identify associated non-verbal learning disorders, as well as language or mathematics-related skills. Furthermore, as few or no gross motor skill disorders were found to be characteristic of VSC dyspraxia, this means that gross motor disorders are not necessarily associated with dyspraxia. The dissociation of such comorbid disorders was made possible because of the systematic investigation of different cerebral functions from a neuro-psychological, neuro-psychomotor and neuro-visual viewpoint on a sample of children enrolled with strict inclusion criteria, hence the need for a multi-dimensional or multi-level assessment of these children [9,10].

Ideomotor patients appear more alike compared to VSC or MX patients, and they are impaired on fewer tasks overall. From a clinical perspective, it is interesting to note that misclassification was only observed for a VSC patient (considered as MX by the PLS classifier). A closer inspection of his medical record further indicated that he suffered from a discrete hemiplegia implying left dysadiadochokinesis, impaired digital praxia, but with normal visual perception.

Hopefully, isolating relevant items among a large set of critical indicators of impaired visuo-motor and cognitive performance might further help to circumvent the lack of consensus around the characterization of dyspraxia subtypes, whether we rely on DSM-IV-R criteria or on the existing literature, see [10] for a review. This will also prove useful for the practician as short and targeted assessment is needed, due to limited resources and time in applied clinical settings. In this regard, the present study suggests that less than 15 skills need to be assessed in order to provide a specific and sensitive diagnostic of DCD subtypes, although the data-driven approaches used here might not fully account for the complexity of skill acquisition or learning process in the target population. Random forests and PLS discriminant analysis were used to reduce the number of relevant features while maximizing the discrimination between given DCD subtypes. As such, they were shown to perform correctly on this particular dataset, and results were consistent with earlier inferential clinical analysis. Whenever more fine hypotheses are to be tested, it makes more sense to turn to methods that allow more flexible modeling of the covariance structure and provide associated tests of hypothesis or pointwise estimation. Of course, the extent to which those results might generalize beyond the sample enrolled in this study is a critical issue. While our methodology was devised so as to limit the risk of overfitting during model selection, our low sample size offers only a limited way to investigate model performance and cluster stability. An external validation study with a larger sample of children, free of any comorbidities, would be needed to confirm the relevance of the highlighted markers.

However, such results could be used to drive more focused investigations of motor control and sensorimotor integration in DCD children; this potentially includes the collection of physiological and cognitive measures when children perform controlled motor tasks, analysis of eye movements dynamics and eye-hand coordination, longitudinal follow-up, etc. As pointed out in the introduction, there is a need for a fairly extensive assessment of different cerebral functions, or a multi-level approach of assessment as suggested by Wilson [9].

## Conclusions

Multidimensional assessment of learning disabilities appears of great interest for the medical community. The statistical analysis of such multivariate and possibly irregular (i.e., few observations, high number of variables) datasets is challenging, but ensemble methods and dimension-reduction techniques can be successfully used to screen variables of interest and assess groupwise clustering profile.

In a sample of 63 children diagnosed as suffering from developmental dyspraxia, these methods provide a concise depiction of two types of pure dyspraxia (ideomotor and visual-spatial/visual-constructional) that are well characterized in the visual-spatial and visual-motor domain, and a third type of dyspraxia (mixt dyspraxia) which features specific comorbidities in addition to impairments shared with the two other types.

## Abbreviations

SITA, Sitting alone; CRAW, Crawling; WALK, Walking alone; FISE, First sentences (language); OTRH, Otorhinolaryngologia; VISR, Visual refraction; LEBL, Lego blocks; PUZL, Puzzles; ARTH, Arithmetic; READ, Reading/spelling; HAWR, Hand writing; DYGR, Dysgraphia; HYPT, Hypotonia; MOPA, Motor pathway; SYNK, Synkinesis; DYSD, Dysadiadochokinesis; STDT, Standing tone; DIPR, Digital praxia; BIDX, Bimanual dexterity; PRSL, Praxia slowness; IMOG, Imitation of gestures; OROP, Orofacial praxia; DRES, Dressing skill; DIPE, Digital perception; VISP, Visual perception; STAB, Static balance; DYNB, Dynamic balance; CULL, Coordination between upper and lower limbs; POSC, Postural control; HLUL, Homogeneity tonic laterality upper/lower limbs; HMLS, Homogeneity manual laterality spontaneous psychomotor; HULU, Homogeneity usual laterality upper/lower limbs; MAND, Manual dexterity; BSPI, Body spatial integration; RHYA, Rhythmic adaptation; VIMI, Visual motor integration; VISS, Visual spatial structuration; VISC, Visual spatial constructional; EXEF, Executive function; AUDM, Auditivo memory; WRKM, Work memory; KINM, Kinaesthetic memory (perception); VISM, Visual spatial memory; AUDA, Auditivo attention; VISA, Visual spatial attention; HYPK, Hyperkinesia; HORP, Horizontal pursuit; VERP, Vertical pursuit; VEPN, Visual evocated potentials (neurovisual).

## Competing interests

The authors declare that they have no competing interests.

## Authors’ contributions

CL designed the statistical study, performed the analysis of data and contributed to their interpretation. LVD was in charge of data collection and clinical assessment. LVD, BG, and BF have made substantial contributions to the interpretation of the data and writing of the manuscript. All authors read and approved the final manuscript.
